# Dynamics of Mitochondrial DNA Copy Number and Membrane Potential in Mouse Pre-Implantation Embryos: Responses to Diverse Types of Oxidative Stress

**DOI:** 10.3390/genes15030367

**Published:** 2024-03-16

**Authors:** Yasmyn E. Winstanley, Jun Liu, Deepak Adhikari, Macarena B. Gonzalez, Darryl L. Russell, John Carroll, Rebecca L. Robker

**Affiliations:** 1 Robinson Research Institute, School of Biomedicine, The University of Adelaide, Adelaide, SA 5005, Australia; yasmyn.winstanley@adelaide.edu.au (Y.E.W.);; 2 Development and Stem Cells Program, Department of Anatomy and Developmental Biology, Monash Biomedicine Discovery Institute, Monash University, Clayton, VIC 3800, Australia

**Keywords:** mtDNA copy number, mitochondrial membrane potential, mitochondrial dysfunction, inner cell mass

## Abstract

Mitochondria undergo a myriad of changes during pre-implantation embryo development, including shifts in activity levels and mitochondrial DNA (mtDNA) replication. However, how these distinct aspects of mitochondrial function are linked and their responsiveness to diverse stressors is not well understood. Here, we show that mtDNA content increased between 8-cell embryos and the blastocyst stage, with similar copy numbers per cell in the inner cell mass (ICM) and trophectoderm (TE). In contrast, mitochondrial membrane potential (MMP) was higher in TE than ICM. Culture in ambient oxygen (20% O_2_) altered both aspects of mitochondrial function: the mtDNA copy number was upregulated in ICM, while MMP was diminished in TE. Embryos cultured in 20% O_2_ also exhibited delayed development kinetics, impaired implantation, and reduced mtDNA levels in E18 fetal liver. A model of oocyte mitochondrial stress using rotenone showed only a modest effect on on-time development and did not alter the mtDNA copy number in ICM; however, following embryo transfer, mtDNA was higher in the fetal heart. Lastly, endogenous mitochondrial dysfunction, induced by maternal age and obesity, altered the blastocyst mtDNA copy number, but not within the ICM. These results demonstrate that mitochondrial activity and mtDNA content exhibit cell-specific changes and are differentially responsive to diverse types of oxidative stress during pre-implantation embryogenesis.

## 1. Introduction

Oocyte mitochondria provide essential energy for pre-implantation embryo development [1,2] and are increasingly recognized for important roles in zygotic epigenetic reprogramming [3,4]. A unique feature of mitochondria is that they possess their own genome (mtDNA), which expresses gene products involved in creating a membrane potential gradient and cellular energy. The importance of mitochondrial function extends far beyond their biosynthetic and bioenergetic roles, with mitochondria now recognized as critically important signaling organelles that rapidly respond to changes within the cell or its surrounding environment [5]. Following fertilization, sperm mitochondria are degraded and, thus, mitochondria are inherited solely through the maternal lineage, meaning all mitochondria (and mtDNA) in offspring are derived from those in the mature oocyte. Importantly, however, the mitochondria of oocytes and embryos are sensitive to physiological cues and are highly susceptible to endogenous and external stressors, and mitochondrial signals are emerging as important epigenetic mechanisms by which offspring phenotypes are inherited [6,7,8]. Thus, oocyte mitochondria quality and quantity are of utmost importance to embryonic development and postnatal health.

During pre-implantation development, mitochondria undergo highly dynamic changes, including intracellular re-localization, biogenesis, and profound morphological changes reflective of a bioenergetic shift from pyruvate to glucose metabolism at the blastocyst stage [9]. Mitochondrial membrane potential (MMP), a key indicator of oxidative phosphorylation and, therefore, overall mitochondrial activity, is known to fluctuate during pre-implantation development, reaching the highest levels in the blastocyst [10], coincident with the metabolic shift. Mitochondrial DNA (mtDNA), distinct from the nuclear genome, is circular and consists of over 16 kb, which encode 37 genes, including multiple gene products that are essential for oxidative phosphorylation [11]. Further, each mitochondrion can contain multiple copies of mtDNA, with the total mtDNA copy number in mammalian MII oocytes in excess of 200,000 copies [12]. Mitochondrial DNA copy numbers show defined patterns following fertilization. As the zygote undergoes cleavage, the mitochondria and mtDNA inherited from the oocyte are segregated into the blastomeres, meaning that the mtDNA copy number per cell decreases with each cell division during pre-implantation development [13]. The total mtDNA copy number in pre-implantation embryos is fairly well characterized and known to fluctuate, in some cases decreasing slightly during the early cleavage stages after fertilization, before increasing at the blastocyst stage [14]. However, the lowest per-cell mtDNA copy number is achieved in the ICM of the blastocyst. These low copy numbers are maintained in the ICM while replication is upregulated in the trophectoderm (TE) [13,14,15,16]. The quantity and quality of mtDNA present within the inner cell mass (ICM) of the blastocyst, understood to be the mtDNA set-point, is fundamentally important for subsequent development, as these mtDNA form the founder population of mtDNA that is replicated in all offspring somatic cells [12,17]. However, the impact of mitochondrial dysfunction on ICM mtDNA levels, which determines the set-point and is critical for cellular differentiation [17], is not well understood.

The mitochondria of pre-implantation embryos are known to be responsive to environmental influences, such as in vitro culture, oxidative stress, and maternal physiology; for instance, obesity and age, which are major contributors to human infertility. Mitochondria quality and function is well established as a key determinant in successful embryo development [2]; therefore, it is important to understand how mtDNA changes during pre-implantation development and whether mtDNA copy numbers are also responsive to these environmental influences. Mitochondrial dysfunction is commonly seen in oocytes and embryos derived from obese or aging females, specifically abnormal metabolism and reduced membrane potential (MMP) [18,19], as well as an increased mtDNA mutation load [19,20]. Further, blastocysts derived from oocytes of obese mice have a reduced mtDNA copy number that is maintained in fetal tissues [19]. Often, in vitro fertilized embryos are cultured in atmospheric conditions, namely 20% oxygen, which is significantly different to the physiological concentrations within the female reproductive tract, estimated to be around 5% [21]. Culture of embryos in atmospheric oxygen conditions has been widely associated with abnormal mitochondria morphology, decreased membrane potential and ATP production [22], and poorer embryo and pregnancy outcomes [23,24,25,26] than those cultured in low oxygen (5%). Thus, mitochondrial defects with obesity, aging, and oxidative stress are well documented; however, it is not clear how these impact mtDNA replication in the blastocyst, specifically the ICM, which goes on to form the fetus.

This study characterized oocyte and embryo mitochondrial activity and mtDNA content throughout pre-implantation development. In addition, responses to four different types of mitochondrial dysfunction (two induced (chemical exposure and high oxygen culture) and two physiological (advanced maternal age and maternal obesity)) were analyzed to elucidate the potential relationships between mitochondrial dysfunction, ICM mtDNA content, and embryo development.

## 2. Materials and Methods

### 2.1. Animals, Hormone Treatment, and Drug Administration

All animal experiments were approved by the University of Adelaide Animal Ethics Committee (Approval Nos. M-2017-116 and M-2018-121) or Monash University Animal Ethics Committee (ID 15094) and conducted in accordance with the Australian Code of Practice for the Care and Use of Animals for Scientific Purposes. Female mice at 6–7 weeks of age and males at 6–8 weeks of age were obtained from the Western Australian Animal Resources Centre (C57BL/6JArc) or the University of Adelaide Laboratory Animal Services (CBA/CaH and CBA F1 (derived from CBA/CaH × C57/BL6Arc)). Mice were maintained in 12 h/12 h light/dark conditions and provided water and 10% fat rodent chow ad libitum.

*PhAM*^loxP/loxP^ mice (Jax mice stock No: 018385) [27] were crossed with transgenic mice that carried Gdf-9 promoter-mediated Cre recombinase (on a C57BL/6J background) [28]. After multiple rounds of crossing, homozygous mutant female mice expressing a mitochondrial-specific version of Dendra2 green/red photo switchable monomeric fluorescent protein exclusively in oocytes (*PhAM*^loxP/loxP^; *Gdf9*-Cre; hereafter Dendra) were obtained.

Obese or reproductive aged female mice, as well as lean young controls, were generated from the same colony (C57BL/6JSfdAnu-Alms1bbb/Apb mouse strain, maintained as heterozygous breeding pairs), termed “Blobby” mice, and fed an identical standard chow diet, as in [19,29]. Obese mice were homozygous (bbb/bbb) for the ‘Blobby’ mutation of the Alms1 gene, which results in hyperphagia and profound obesity even when maintained on a standard mouse chow diet [19]. Females were deemed obese when they weighed at least 36 g, which occurred at 4–5 months of age, and wild-type littermates were used in parallel as lean controls. Reproductive aged females were wild-type or heterozygous (+/+ or bbb/+) at 12 months of age, and young females (3–4 months old) were used in parallel as young controls.

Rotenone (#R8875) was sourced from Sigma (St. Louis, MO, USA) and a rotenone diet was prepared and used, as in [30]. Specifically, the control diet was a modified diet of AIN-93G (TD.97184), and the rotenone diet was formulated at 150 ppm rotenone in the TD.97184 meal diet by Teklad/Envigo, (Madison, WI, USA) exactly as in [30]. CBA F1 mice were randomly assigned to the control or rotenone diet for three weeks, as per our previous experiments [29]. This dose of rotenone is demonstrated to decrease Complex I- and Complex II-driven respiration without altering oxidative phosphorylation subunit abundance or causing overt physiological effects [30].

Female mice were administered pregnant mare serum gonadotropin (PMSG, #493-10, Lee BioSolutions, Maryland Heights, MO, USA) at 5 IU/12 g body weight, followed by an equivalent dose of human chorionic gonadotropin (hCG, Pregnyl, Marck Sharp & Dohme, Macquarie Park, NSW, Australia) 47.5 h later, each via intraperitoneal (ip) injection.

### 2.2. Derivation of Oocytes, Early Embryos, and Blastocysts

Germinal vesicle (GV) oocytes were collected 45 h after PMSG administration, and Meiosis II (MII) oocytes were collected 15.5 h post-hCG administration. Mice were culled via cervical dislocation, and ovaries and oviducts were collected and placed in pre-warmed (37 °C) αMEM-HEPES handling media. GV oocytes were isolated by puncturing large antral ovarian follicles and attached granulosa cells were removed by aspiration using a glass pipette pulled to an appropriate diameter. Ovulated cumulus oocyte complexes (COCs) were isolated by opening the oviducts using a 30 G needle. For collection, cumulus cells were completely removed from MII oocytes via treatment with hyaluronidase (Seikagaku, Chiyoda, Tokyo, Japan, #100741) for 5–10 min at 37 °C, followed by aspiration using a glass pipette pulled to an appropriate diameter.

Embryos were generated at precise stages of development via in vitro fertilization (IVF). Briefly, ovulated COC clusters were gently washed twice in pre-warmed (37 °C) wash media (Vitro Wash; Cook Australia, Brisbane, QLD, Australia), before being placed in a 100 μL fertilization drop containing the equivalent of 10 μL of capacitated sperm from a male of proven fertility (referred to as ‘fertilization time’), before being returned to the incubator (37 °C, 5% O_2_, 6% CO_2_) for 4 h. Fertilized oocytes were then cleaned of all excess sperm and cumulus cells via gentle aspiration, and only those with 2PN or 2PB were deemed to have been fertilized and were transferred to a culture dish containing cleave media (Vitro Cleave; Cook Australia, Brisbane, QLD Australia, 10 embryos per 20 μL of cleave media drop) and returned to the incubator. A subset of zygotes was collected at 6 h post-fertilization time for analysis, and the rest were incubated until 24 h post-fertilization time. At this time, the number of embryos that successfully reached the 2-cell stage were scored. For high-oxygen (20% O_2_) experiments, the fertilization incubation and subsequent culture in cleave media were conducted at 37 °C, 20% O_2_, and 6% CO_2_.

The number of ovulated oocytes per mouse and embryo on-time development were monitored in every experiment to ensure collection of embryos at the precise stage. The 4-cell embryos were collected at 39 h post-fertilization (early day 3 of culture), 8-cell embryos at 55 h (late day 3 of culture), morula at 77 h (day 4 of culture), and blastocysts at 96 h (day 5 of culture, equivalent to time of implantation in vivo) post-fertilization time. Morulae collected for analysis were all of a similar cell number, and blastocysts were collected at 100 h post-fertilization time and were all at a similar developmental stage (late-expanded or early hatching) and morphologically normal.

To obtain in vivo 2-cell, 8-cell, or blastocyst embryos, female mice were mated with male mice at the time of the hCG injection, and female mice were culled by cervical dislocation at day E1.5, E2.5, or E3.5 for 2-cell, 8-cell, or blastocyst embryos, respectively. Oviducts (2-cell and 8-cell) and uteri (blastocysts) were collected as previously described and embryos were retrieved.

### 2.3. Isolation of Blastocyst Cell Populations

Collection of paired inner cell mass (ICM) and trophectoderm (TE) cell populations was performed by manual dissection. The zona pellucida was removed using Acid Tyrode’s Embryomax solution (Merck Millipore, Burlington, MA, USA, #MR-004D) and blastocysts were transferred to individual pre-warmed (37 °C) drops of αMEM handling media with 1% FCS in a 50 × 9 mm petri dish (Falcon, #351006, VWR, Tingalpa, QLD, Australia). Blastocysts were manually separated into their two cell populations using the Eppendorf TransferMan 4r system, and a standard holding pipette with a 15 μm inner diameter and 120 μm outer diameter (The Pipette Company, #LHC-ID15, CooperSurgical, Sydney, NSW, Australia) and a biopsy pipette with a 30 μm tip (OD) and 90° bevel (The Pipette Company, #LBC-OD30-BA90). Cell populations were separated and frozen individually per blastocyst but were paired for analysis.

Immuno-surgery was used to isolate a purified ICM population. Blastocysts had their zona pellucidae removed via treatment with Acidic Tyrode’s Embryomax solution, were washed twice in cleave media, and were transferred to cleave media containing 20% heat-inactivated rabbit anti-mouse serum (Sigma, St. Louis, MO, USA, #M5774). Following a 1 h incubation (37 °C, 5% O_2_, 6% CO_2_), blastocysts were washed three times in cleave media and transferred to cleave media containing 20% guinea pig serum (Sigma, #G9774) and incubated for 15 min. Blastocysts were washed three times in cleave media and incubated for a further 30 min in this media. Lysed trophectoderm (TE) cells were removed by gently aspirating ICMs in a finely drawn glass pipette.

### 2.4. Sample Collection and DNA Extraction

Individual oocytes or embryos for qPCR analysis were washed three times in 1× phosphate buffered saline (PBS) containing 1 mg/mL of polyvinylpyrrolidone (PVP, Sigma), and transferred in 1 μL to a 0.5 mL PCR tube, snap frozen in liquid nitrogen (LN2), and stored at −80 °C until use. DNA was extracted from oocytes and embryos by adding 9 μL of lysis buffer (50 mM Tris-HCl, pH 8.0, 1 mM EDTA, 200 μg/mL Proteinase K, 0.5% Tween-20) to each sample (final volume of 10 μL) and heating to 55 °C for 2 h, followed by 95 °C for 10 min. Samples were cooled to 4 °C and briefly centrifuged. ICM samples were diluted with an additional 10 μL of sterile H_2_O to yield a total volume of 20 μL. Oocytes and embryo samples had 1 μL of lysis product removed and added to 9 μL of sterile H_2_O for mtDNA analysis due its abundance, and the rest was used for Rn18S (reference gene) analysis.

### 2.5. Quantitative PCR (mtDNA, Rn18S, and SRY)

To generate ‘calibrator’ DNA for use as a standard within every assay, a whole ovary from a 6-week-old PMSG- and hCG-stimulated CBA female mouse was collected, total DNA was extracted using a QIAamp DNA Micro Kit (Qiagen, Hilden, Germany), as per the manufacturer’s instructions for ‘Isolation of Genomic DNA from Tissues’, and it was eluted in a final volume of 50 μL of sterile H_2_O. The DNA concentration and purity were quantified by a NanoDrop One Microvolume UV-Vis Spectrophotometer and diluted to 2.5 ng/μL.

qPCR was performed using Power SYBR Green PCR Master Mix (Applied Biosystems, Foster City, CA, USA, #4367659), standard MicroAmp Optical 96-well Reaction Plates (Life Technologies, Carlsbad, CA, USA, I bel #4306737), and custom-made primers (Sigma), using an Applied Biosystems 7900 HT Fast Real-Time PCR system. Cycling conditions for all primers were set at 95 °C for 10 min, followed by 40 cycles of 95 °C for 10 s, 60 °C annealing for 30 s, and extension at 72 °C for 30 s, followed by standard melt curve cycling. Two replicate reactions were performed for each sample and each primer pair, using 2 μL of sample per reaction. mtDNA sequences were: mtDNA forward 5′-CGT TAG GTC AAG GTG TAG CC-3′, and mtDNA reverse 5′-CCA GAC ACA CTT TCC AGT ATG-3′ [19]. Reference gene primer sequences were: Rn18S forward 5′-AGA AAC GGC TAC CAC ATC CAA-3′, and Rn18S reverse 5′-CCT GTA TTG TTA TTT TTC GTC ACT ACC T-3′ [31].

For mtDNA analysis, the final reaction for each well consisted of 10 μL of Power SYBR Green PCR Master Mix, 0.2 μL each of forward and reverse primers (stock at 10 μM), 2 μL of sample or standard, and sterile H_2_O to a final volume of 20 μL. The total mtDNA copy number was quantified using the standard curve method, as previously described [19,32]. A standard curve ranging from 10^7^ to 10^1^ copies was generated, and standard curve correlation coefficients were consistently greater than 0.98. The total mtDNA copy number per sample was calculated according to the generated equation for the Ct value against the copy number for the corresponding standard curve, as detailed in [19].

For Rn18S analysis, the final reaction for each well was 10 μL of Power SYBR Green PCR Master Mix, 0.5 μL each of forward and reverse primers (stock at 10 μM), 2 μL of sample, and sterile H_2_O to a final volume of 20 μL. Calibrator DNA was included on every plate.

mtDNA relative to the cell number was calculated using the 2^−ΔΔCt^ method, where the ΔCt of the calibrator (mtDNA Ct—Rn18S Ct) was subtracted from the ΔCt of the sample (mtDNA Ct—Rn18S Ct) to yield the ΔΔCt.

Embryos that underwent sex determination first underwent analysis for mtDNA and Rn18S. qPCR for the SRY sequence was performed, as previously described [33]. One reaction was performed for each sample, using 4 μL of sample. The final reaction for each sample consisted of 10 μL of Power SYBR Green PCR Master Mix, 0.5 μL each of forward and reverse primers (stock at 10 μM, forward primer 5′-AAG CGC CCC ATG AAT GCA TT-3′, and reverse primer 5′-TCC CAG CTG CTT GCT GAT CT-3′), 4 μL of sample, and sterile H_2_O to a final volume of 20 μL. To confirm the qPCR product size, reactions were run on 4% agarose gel. Male embryos had a distinct band at 105 bp.

### 2.6. Transfer of Blastocyst Embryos to Pseudo-Pregnant Female Mice and Fetal Tissue Analysis

Embryos were vitrified at the morula stage and later thawed, cultured to blastocysts, and transferred to pseudo-pregnant female mice (14 embryos of one type to each mouse (7 embryos per side)). Mice were humanely killed by cervical dislocation on day 18.5 of pregnancy. Fetuses were humanely euthanized, and tissues were collected and individually snap frozen in liquid N_2_ and stored at −80 °C until use.

Fetal heart and liver tissues were lysed overnight at 55 °C with constant shaking (100 rpm) in 250 μL of lysis buffer (20 mM EDTA, pH 8.0, 50 mM Tris pH 8.0, 120 mM NaCl, 1% SDS) with 5 μL of Proteinase K (10 mg/mL). The following morning, 250 μL of 4 M ammonium acetate was added, briefly vortexed, then incubated for 15 min at RT with shaking, and then a further 10 min without. Samples were centrifuged at 10,000 rpm for 10 min, and 400 μL of supernatant was removed and added to a clean tube. Then, 800 μL of 100% ethanol was added and briefly vortexed prior to centrifugation at 10,000 rpm for 8 min to pellet the DNA. Ethanol was removed and the pellet was washed using 500 μL of 70% ethanol. Excess ethanol was then removed, and DNA was re-suspended by incubation at 55 °C with shaking (100 rpm) in 300 μL of sterile H_2_O. DNA was quantified using a ThermoFisher Nanodrop One UV-Vis spectrophotometer and diluted to 4 ng/μL with sterile water for use. The relative mtDNA content was analyzed in fetal tissues via qPCR, as described above. mtDNA and reference gene (Rn18S) primer sequences and cycling conditions were as above. The final reaction for each well was 10 μL of Power SYBR Green PCR Master Mix, 0.2 μL each of forward and reverse primers (stock at 10 μM), 2 μL of sample, and sterile H_2_O to a final volume of 20 μL, and two replicate reactions were performed for each sample. mtDNA relative to reference gene copies was calculated using the formula: 1/(mtDNA Ct/reference Ct).

### 2.7. TMRM Staining of Mitochondrial Membrane Potential

Embryos at the desired stage were incubated in 50 nM of TMRM for 30 min at 37 °C in the dark, then washed in fresh medium 3 times before imaging. For timelapse imaging, 8-cell embryos (fertilized in vivo and flushed from the tract) were plated in 5 μL droplets of SAGE One-Step embryo culture medium (CooperSurgical Fertility and Genomic Solutions, Sydney, NSW, Australia) on a glass-bottom dish covered by mineral oil. Embryos were imaged using a laser-scanning confocal system (SP8, Leica, Wetzlar, Germany) with a stable incubation chamber set at 37 °C in 5% CO_2_ and 20% O_2_. A 552 nm laser line was used to image the TMRM signal. Images were acquired every 30 min.

Live embryos labeled with TMRM were imaged at 37 °C in a glass-bottom dish using a laser-scanning confocal system (SP8, Leica). A 488 nm laser line and a 495–523 nm bandpass filter were used to image the Dendra signal. A 552 nm laser line and a 563–627 nm bandpass filter were used to image the TMRM signal. The line average was set at 4 times for high-quality images. Ratiometric analysis was performed using ImageJ software (version 2.0.0). Briefly, Dendra (green) and TMRM (red) channels of the confocal images were separated and converted to 32-bit images, and the background of each channel was set to Not a Number (NaN) using thresholding, which was determined visually. The same threshold was applied to other images from the same experiment. Then, the TMRM fluorescence value was divided by the Dendra fluorescence value pixel-by-pixel using the Image Calculation function in ImageJ. The calculated ratio image is shown by a preset lookup table in ImageJ called 16_colors, which shows higher values with a warmer color.

### 2.8. MitoSOX Red (MSR) Staining

Zygotes were collected and incubated for 20 min at 37 °C in handling media containing 60 µg/mL of BSA, 1 mg/mL of PVP, and 5 µM of MitoSOX Red (Invitrogen, Carlsbad, CA, USA, #M36008), with Hoescht-3342 (1 µg/mL). Samples were briefly washed in handling media before being mounted in pre-warmed handling media (containing BSA and PVP, as above) and imaged using an Olympus FV3000 Confocal Microscope at 60× magnification, with a single z-plane chosen for each zygote where the diameter was largest. Images were acquired using the same confocal microscope settings, with the operator blinded to the treatment group. Representative images were shown.

### 2.9. Statistical Analysis

The results are presented as mean ± SEM. On-time development data are displayed as mean ± SEM overlaid on a violin plot to illustrate the distribution of data points. All data points represent independent biological replicates. Statistical analysis was performed using GraphPad Prism version 8 for Windows (GraphPad Software Inc., La Jolla, CA, USA) and SPSS Statistics 26 (IBM, Armonk, NY, USA). The paired two-tailed *t*-test, unpaired two-tailed *t*-test, one-way and two-way analysis of variance (ANOVA, comparison of means), and linear-mixed-effects models were used as indicated, and statistical significance was considered at *p*-value < 0.05.

## 3. Results

### 3.1. Mitochondrial DNA across Pre-Implantation Development and in Blastocysts

To measure mitochondrial DNA (mtDNA) in individual oocytes and embryos, a qPCR assay was developed to quantify both the total mtDNA copy number and the relative copy number per cell (Figure S1). Primers specific to the mitochondrially encoded 12S rRNA gene were used to detect mtDNA and, in order to allow direct comparison of embryos with different numbers of cells (i.e., zygotes, 8-cells, and blastocysts), primers for a multi-copy nuclear genome reference sequence (Rn18S, [31]) were also incorporated. Thus, changes in mtDNA could be expressed relative to the nuclear DNA content of each embryo, accounting for changes in the cell number (Figure S1).

**Figure 1:**
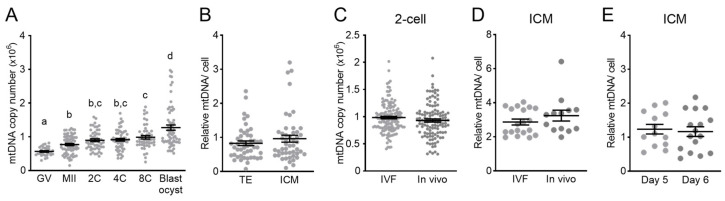
mtDNA copy number during mouse pre-implantation development. (**A**) Total mtDNA copy number throughout a pre-implantation embryo development time course of IVF-derived embryos (minimum of n = 29 oocytes or embryos analyzed at each developmental stage). (**B**) Relative mtDNA content per cell in the trophectoderm (TE) and inner cell mass (ICM) micro-dissected from the same blastocysts (n = 48). (**C**) mtDNA copy number in 2-cell embryos fertilized and cultured in vitro (IVF, n = 141) or fertilized in vivo and collected 40 h following hCG (n = 119). (**D**) Relative mtDNA content per cell in the ICM from blastocysts fertilized and cultured in vitro (IVF, n = 13) or fertilized in vivo and collected 93 h following hCG (n = 19). (**E**) Relative mtDNA content per cell in the ICMs of blastocysts on day 5 (n = 13) or day 6 (n = 16) of culture. Data were analyzed using a linear-mixed-effects model (**A**), where different lowercase letters indicate statistical significance between groups of at least *p* < 0.05, the paired *t*-test (**B**), or the unpaired two-tailed *t*-test (**C**–**E**).

This assay was used to measure the mtDNA copy number in CBAF1 mouse oocytes and embryos at multiple timepoints throughout pre-implantation development to provide a comprehensive analysis of mtDNA kinetics (Figure 1A). The mtDNA copy number increased between GV oocytes and ovulated MII oocytes collected 16 h later (Figure 1A). In a separate cohort, the mtDNA copy number was not different between MII oocytes (1,012,880 ± 27,214, n = 113) and 1-cell zygotes (1,029,255 ± 22,140, n = 133) collected 6 h following fertilization (*p* = 0.67). From the MII oocyte stage through the 8-cell stage of development, the mtDNA copy number remained constant (Figure 1A). Between the 8-cell and blastocyst stages, the mtDNA copy number per cell increased (Figure 1A). To verify that these patterns were conserved across mouse strains, the mtDNA copy number was analyzed in oocytes, 2-cell, 4-cell, 8-cell, and blastocyst stage embryos derived from a further 4 different inbred and outbred genetic backgrounds (Figure S2). Embryos from some mouse strains showed a small decline in the mtDNA copy number between fertilization and the 8-cell stage, while others stayed constant (Figure S2B–E), but all mouse strains exhibited the biggest increase in the mtDNA copy number between the 8-cell and blastocyst stages (Figure S2B–E).

Further characterization of mtDNA patterns included comparison of relative mtDNA per cell in the trophectoderm (TE) and inner cell mass (ICM) of the same blastocysts. At 100 h post-fertilization, the mtDNA content was not different between the two cell types (Figure 1B). Since mitochondria are sensitive to the cellular environment, the mtDNA copy number was compared between embryos fertilized in vitro versus in vivo. In vitro fertilization and culture using our standard conditions (clinical-grade media and 5% O_2_ incubators) did not affect the total mtDNA copy number in 2-cell embryos (Figure 1C) or relative mtDNA per cell in the ICM (Figure 1D), indicating that optimized preclinical protocols do not overtly alter mtDNA dynamics during pre-implantation development. Further, an additional day of blastocyst culture (to approximately ~24 h after typical implantation timing) did not affect the ICM mtDNA content (Figure 1E), highlighting the stable nature of mtDNA content within the ICM across this peri-implantation developmental window. Since male (XY) and female (XX) embryos exhibit metabolic differences [34], the mtDNA content was compared in whole blastocysts and ICMs of each sex but was found to have no differences (Figure S3).

Together, these results indicate that mtDNA copy number kinetics during pre-implantation development are relatively conserved across mouse strains, with the total copy number consistently increasing slightly from the 8-cell to blastocyst stage. Further, the culture conditions used here did not perturb the embryo mtDNA copy number when compared to those developing in vivo, and embryo sex did not differentially regulate the blastocyst or ICM mtDNA content.

### 3.2. Mitochondrial Membrane Potential across Pre-Implantation Development and in Blastocysts

**Figure 2:**
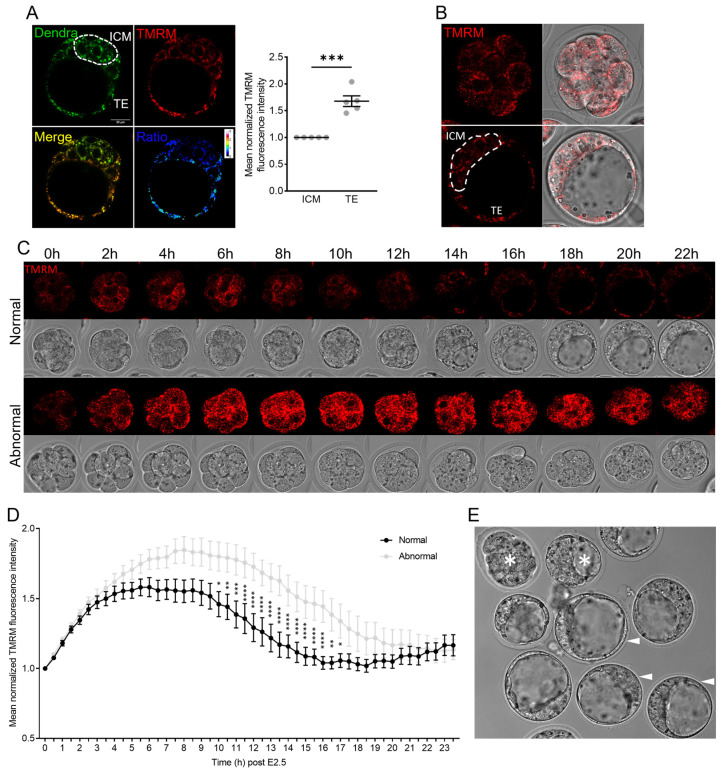
Mitochondrial activity from the 8-cell to blastocyst stage differs with embryo developmental competency. (**A**) In vivo blastocysts from mito-Dendra mice were labeled with TMRM, and the TMRM/Dendra ratio of the ICM and TE was determined (n = 5). (**B**) In vivo fertilized 8-cell embryos (upper panel) were loaded with TMRM and cultured to the blastocyst stage (lower panel). (**C**) Representative timelapse images of an 8-cell embryo that exhibited normal development and one that exhibited abnormal development across the 24 h culture period. TMRM (red) is shown in the top panel and brightfield in the bottom. (**D**) Mean normalized TMRM intensity of embryos that developed into normal (n = 23) versus abnormal (n = 22) blastocysts. (**E**) Brightfield image of embryos after the 24 h timelapse confocal imaging. Normally developed embryos are indicated by the arrow heads, while abnormal embryos are indicated by the asterisks. Data were analyzed via the paired *t*-test (**A**) or two-tailed *t*-test (**D**): * *p* < 0.05, ** *p* < 0.01, *** *p* < 0.001, and **** *p* < 0.0001.

Mitochondrial membrane potential (MMP), reflective of energy production, was measured to compare to mtDNA kinetics during pre-implantation development. TMRM potentiometric dye (a cell-permeant label that accumulates in active mitochondria with intact membrane potential [35]) was used as an indirect measure of MMP. In zygotes, TMRM was highly responsive to mitochondrial disruption by FCCP (mitochondrial uncoupler) and rotenone (Complex I inhibitor; Figure S4). Initially, embryos of mice expressing mitochondrial Dendra (a mitochondrial-specific version of Dendra2 green/red photo switchable monomeric fluorescent protein [36]) were stained with TMRM to co-localize active mitochondria within the ICM and TE. TMRM staining of blastocyst stage embryos flushed from the uteri of Dendra mice at E3.5 showed that MMP was higher in TE cells than in ICM cells (Figure 2A).

To explore this differential result further, MMP was monitored from the 8-cell to blastocyst stage by culturing 8-cell embryos (fertilized in vivo and flushed from the tract) in media containing TMRM and following each via timelapse imaging (Figure 2B). TMRM intensity increased during the first few hours of culture following the 8-cell stage (Figure 2C,D). At the end of culture, embryos were categorized into those that formed normal blastocysts or those that failed to develop normally (i.e., arrested at the 8-cell or morula stage or formed an abnormal blastocoel; Figure 2C). Embryos that formed normal blastocysts had relatively stable TMRM staining that began to decline around the time of morula formation and up to the blastocyst stage (Figure 2C,D). In contrast, abnormal embryos had an increased mean TMRM intensity that remained significantly higher than that of the normal embryos from the morula stage until the time the blastocoel cavity began to form (Figure 2C,D). These results indicate a link between poor embryo development and dramatically upregulated MMP. We hypothesized that the high proportion of abnormal embryos and the dramatically upregulated TMRM intensity in these embryos was due to their exposure to atmospheric oxygen conditions during culture and timelapse imaging.

### 3.3. Mitochondrial Membrane Potential and mtDNA Dysregulated by High-Oxygen Culture

**Figure 3:**
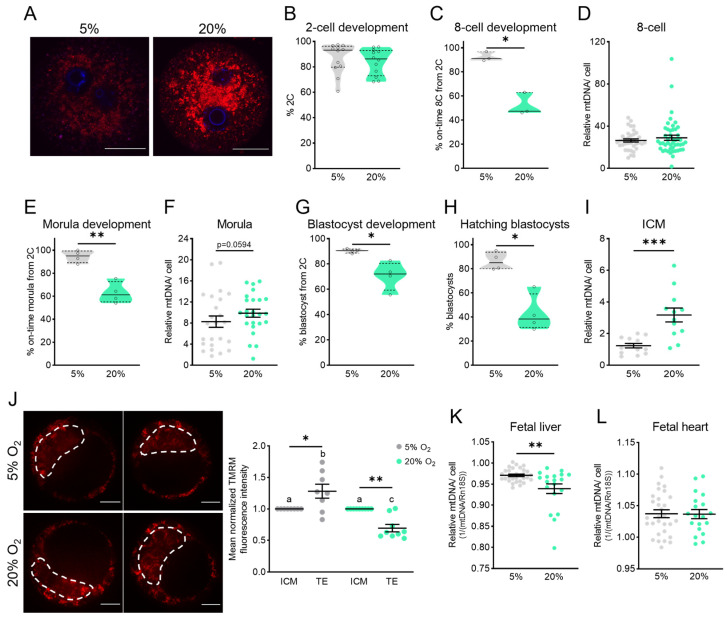
High-oxygen culture delays embryo development and increases ICM mtDNA content. (**A**) mtROS was detected using the MitoSOX Red Superoxide Indicator in zygotes from control (5%) or high-oxygen (20%) culture. Scale bar is 20 µm. (**B**) On-time development to the 2-cell stage (n = 12 independent replicates) of embryos cultured in low (5%) or high (20%) oxygen. (**C**) On-time development to the 8-cell stage (n = 3 independent replicates). (**D**) Total mtDNA copy number in 8-cell embryos (n = 36 at 5% and n = 47 at 20%). (**E**) On-time development to the morula stage (n = 4 independent replicates). (**F**) Total mtDNA copy number in morula embryos (n = 24 at 5% and n = 25 at 20%). (**G**) On-time development to the blastocyst stage (n = 4 independent replicates). (**H**) The proportion of blastocysts that were hatching (n = 4 independent replicates). (**I**) Relative mtDNA per cell in purified ICMs (n = 13 at 5% and n = 12 at 20%). (**J**) In vivo 8-cell embryos were collected and cultured in the presence of TMRM (10 nM) to the blastocyst stage in either 5% O_2_ or 20% O_2_ with TMRM fluorescence in the ICM and TE quantified (n = 8 at 5% and n = 9 at 20%). TE fluorescence normalized to ICM fluorescence. Scale bar is 20µm. (**K**) Relative mtDNA per cell in fetal (18.5 dpc) liver (n = 30 at 5% and n = 19 at 20%) and (**L**) fetal heart (n = 30 at 5% and n = 19 at 20%) following blastocyst stage embryo transfer to identical surrogates. Data were analyzed using the paired *t*-test (**B**,**C**,**E**,**G**,**H**) or unpaired *t*-test (**D**,**F**,**I**,**K**,**L**). (**J**) Data were analyzed via the paired *t*-test (asterisks) and one-way ANOVA, where different lowercase letters indicate statistical significance of at least *p* < 0.05: * *p* < 0.05, ** *p* = 0.0073, and *** *p* ≤ 0.0008.

To directly investigate the impact of high-oxygen culture conditions on mtDNA and MMP during pre-implantation embryogenesis, the effects of high (20%) and low (5%) oxygen conditions on embryo development, mtDNA, and TMRM intensity were compared (Figure 3). High-oxygen culture increased mitochondrial ROS production (Figure 3A) but did not alter the proportion of embryos that successfully developed to the 2-cell stage (Figure 3B); however, the proportion of those 2-cell embryos that successfully developed to 8-cell embryos on time was reduced (Figure 3C). Of those that developed to the 8-cell stage on time, the mtDNA content was not different between the two groups (Figure 3D). The delay in embryo development at the 8-cell stage (Figure 3C) persisted to the morula stage, with a reduced proportion of 2-cell embryos cultured in 20% O_2_ forming morula stage embryos on time (Figure 3E). The mtDNA content was analyzed in on-time morulae and trended toward being higher in those cultured in 20% O_2_ (Figure 3F); however, this was not statistically significant (*p* = 0.0594). High-oxygen culture also reduced the percentage of embryos that developed on time to blastocysts (Figure 3G), as well as the proportion of blastocysts that were hatching (Figure 3H), consistent with previous observations [37]. The mtDNA content in the inner cell mass (ICM) was increased in embryos cultured in high (20%) oxygen compared to those cultured at 5% (Figure 3I).

To investigate the effects of 20% O_2_ on MMP, in vivo fertilized embryos were collected at the 8-cell stage, loaded with TMRM, and cultured in either 5% O_2_ or 20% O_2_ to the blastocyst stage. Embryos cultured at 5% O_2_ exhibited higher TMRM fluorescence in the TE than the ICM (Figure 3J), which was similar to the relatively higher TMRM intensity in TE of the in vivo flushed blastocysts (Figure 2A). Quantification of the TMRM intensity in the ICM and TE populations found that high-oxygen culture disrupted normal mitochondrial bioenergetics in blastocysts, as TMRM intensity in TE was reduced compared to the ICM with high-oxygen culture.

To investigate the implications for subsequent fetal development, embryos cultured in 5% O_2_ or 20% O_2_ were transferred to pseudo-pregnant surrogates, and fetuses were examined on day 18.5 of gestation. Even though equal numbers of blastocysts (i.e., 14 blastocysts) were transferred to each mouse, fewer fetuses were observed in the females that received embryos cultured at 20% O_2_ (29.73% ± 3.924%, n = 4 surrogate females) compared to those cultured at 5% O_2_ (46.88% ± 3.125%, n = 4 surrogate females, *p* = 0.0142), indicative of a reduced implantation potential. Liver and heart tissues were collected from the fetuses and analyzed for mtDNA content. High-oxygen culture until the blastocyst stage was associated with reduced mtDNA content in fetal livers (Figure 3K) but did not alter the mtDNA content in fetal hearts (Figure 3L).

### 3.4. Mitochondrial Disruption (Rotenone, Aging, or Obesity) Prior to IVF Does Not Alter mtDNA in ICM

**Figure 4:**
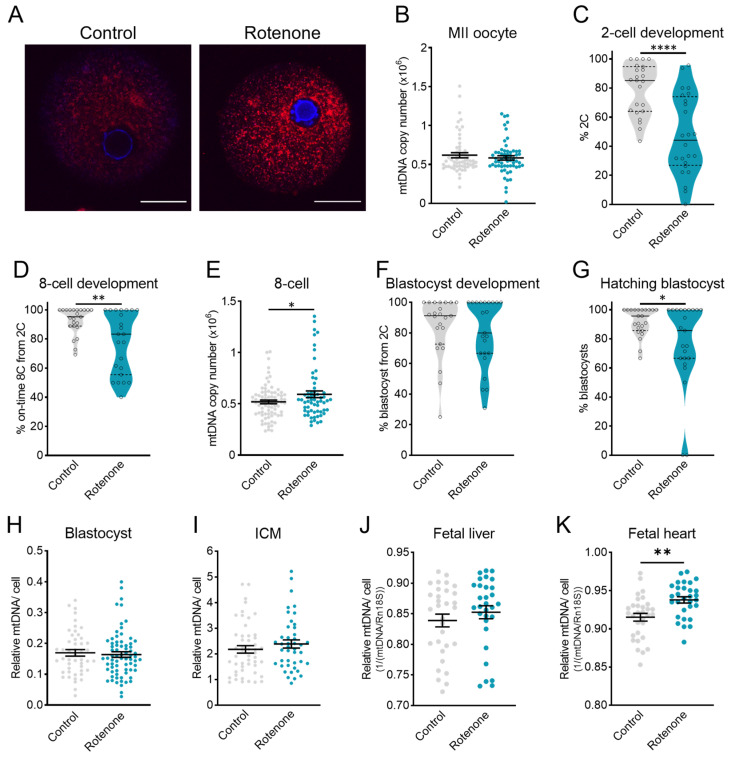
Rotenone-induced mitochondrial dysfunction delays embryo development but does not alter the mtDNA copy number in blastocysts. (**A**) mtROS was detected using the MitoSOX Red Superoxide Indicator in zygotes from control mice or mice exposed to a low dose of rotenone. (**B**) Total mtDNA copy number in MII oocytes from control mice or rotenone-exposed females (n = 59 control and n = 60 rotenone). (**C**) On-time development to the 2-cell stage (n = 23 control and n = 24 rotenone). (**D**) On-time development to the 8-cell stage (n = 23 control and n = 23 rotenone). (**E**) Total mtDNA copy number in 8-cell embryos from control or rotenone-exposed females (n = 80 control and n = 61 rotenone). (**F**) On-time development to the blastocyst stage (n = 23 control and n = 23 rotenone). (**G**) The proportion of blastocysts that were hatching. (**H**) Total mtDNA copy number in blastocysts (n = 49 control and n = 72 rotenone). (**I**) Relative mtDNA per cell in purified ICMs (n = 50 control and n = 43 rotenone). (**J**) Relative mtDNA per cell in fetal (18.5 dpc) liver (n = 31 control and n = 30 rotenone) and (**K**) fetal heart (n = 31 control and n = 30 rotenone) following blastocyst stage embryo transfer to identical surrogates. Each data point for on-time development represents the mean of all embryos from one female mouse. Data were analyzed using the unpaired *t*-test: * *p* < 0.035, ** *p* < 0.003, and **** *p* < 0.0001.

**Figure 5:**
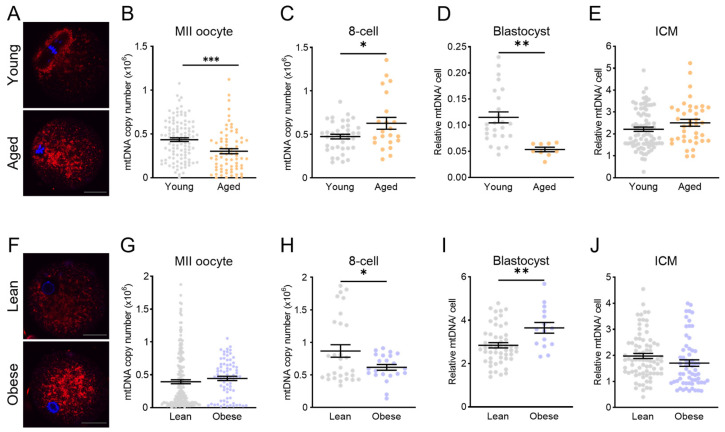
Advanced maternal age and maternal obesity differentially affect blastocyst mtDNA content. (**A**) mtROS was detected using the MitoSOX Red Superoxide Indicator in MII oocytes from young and reproductive aged (12 months) females. (**B**) Total mtDNA copy number in MII oocytes from young or aged (n = 110 young and n = 71 aged) female mice. (**C**) Total mtDNA copy number in 8-cell embryos from young or aged females (n = 39 young and n = 22 aged). (**D**) Total mtDNA copy number in blastocysts (n = 32 young and n = 13 aged). (**E**) Relative mtDNA per cell in purified ICMs (n = 82 young and n = 38 aged). (**F**) mtROS (MitoSOX Red Superoxide Indicator) in zygotes from lean and obese females. (**G**) total mtDNA copy number in MII oocytes from lean (n = 192 oocytes) and obese (n = 75 oocytes) female mice. (**H**) Total mtDNA copy number in 8-cell embryos (n = 29 lean, n = 22 obese). (**I**) Total mtDNA copy number in blastocysts (n = 51 lean, n = 15 obese). (**J**) Relative mtDNA per cell in purified ICMs (n = 79 lean, n = 61 obese). Data were analyzed using the unpaired *t*-test: * *p* < 0.04, ** *p* = 0.0016, and *** *p* = 0.0003.

To more specifically isolate the role of mitochondrial dysfunction on mtDNA levels throughout pre-implantation development, an inhibitor of the function of mitochondrial Complex I, rotenone, was used. Rotenone exposure causes mitochondrial dysfunction by blocking oxidative phosphorylation and reducing ATP synthesis [21], and increasing ROS production [22,23]. Mice were exposed to a low dose of rotenone (150 ppm, shown to elicit a mild inhibition of mitochondrial function [30]) via their diet for three weeks before ovulated eggs were collected and underwent IVF in identical conditions to the controls. Exposure to rotenone resulted in more mitochondrial ROS production (Figure 4A) but did not alter the mtDNA copy number in MII oocytes (Figure 4B). Rotenone exposure affected oocyte competence to fertilize and cleave, as the proportion that successfully developed to 2-cell embryos was reduced compared to the controls (Figure 4C). Rotenone-exposed oocytes also exhibited a decrease in the proportion of 2-cell embryos that reached the 8-cell stage on time (Figure 4D). The total mtDNA copy number was slightly increased in 8-cell embryos derived from rotenone-exposed oocytes (Figure 4E). Despite the reduction in 2-cell and 8-cell development (Figure 4C,D), rotenone exposure did not impact the proportion of 2-cell embryos that successfully developed to blastocysts on time (Figure 4F), indicating accelerated development after the 8-cell stage. However, the proportion of blastocysts that had begun hatching at 96 h after fertilization was reduced (Figure 4G). Interestingly, in the embryos that developed on time to blastocysts, mtDNA in whole blastocysts (Figure 5H) was not different between groups and, specifically, the ICM was not affected (Figure 5I).

Blastocysts from control or rotenone-exposed eggs were transferred to pseudo-pregnant surrogates (14 embryos per female), and fetuses were collected on day 18.5 of gestation and their liver and heart tissues were analyzed for mtDNA content. No differences in implantation potential (ratio of fetuses per embryos transferred) were observed between embryos derived from control (32.29% ± 6.76%, n = 8 surrogate females) or rotenone-exposed females (35.73% ± 5.95%, n = 7 surrogate females, *p* = 0.712). Exposure of oocytes to rotenone did not significantly alter the mtDNA content in fetal livers (Figure 4J) but was associated with an increase in mtDNA content in fetal hearts (Figure 4K).

We next investigated how mitochondrial dysfunction in the physiological contexts of obesity and aging alters the mitochondrial DNA copy number. ROS levels were elevated (Figure 5A), and the mtDNA copy number was lower (Figure 5B) in MII oocytes from reproductive aged females, compared to those from young controls. Embryo on-time development from oocytes of reproductive aged females was not different to young controls (Figure S5A–C). Analysis of 8-cell embryos showed a higher mtDNA copy number in those derived from aged females (Figure 5C); however, at the blastocyst stage, the mtDNA copy number was reduced in whole blastocysts derived from aged females (Figure 5D). The mtDNA content in the ICM, however, was not different in embryos from aged females compared to those from young females (Figure 5E), suggesting that the reduction in the blastocyst mtDNA copy number was due to changes in the trophectoderm cell population.

Higher mitochondrial ROS production was also evident in zygotes derived from obese females (Figure 5F); however, maternal obesity did not influence the mitochondrial DNA copy number in MII oocytes (Figure 5G). Maternal obesity reduced the proportion of oocytes that were able to successfully fertilize and cleave to the 2-cell stage of development (Figure S5D) but did not alter on-time development to the 8-cell or blastocyst stages (Figure S5E,F). The mtDNA copy number in the 8-cell embryos was reduced by maternal obesity (Figure 5H), an opposite trend to what was observed in embryos from reproductive aged females (Figure 5C). The mitochondrial DNA copy number was upregulated in blastocysts from obese females (Figure 5I), again the opposite trend to that in blastocysts from reproductive aged females (Figure 5D). The mtDNA content was not different in the ICM population (Figure 5J), indicating that the upregulation of mtDNA copy numbers in whole blastocysts originates in the trophectoderm. Due to their limited numbers, embryos from these groups were not transferred to surrogates for gestation. Cumulatively, however, these results indicate that even in the absence of morphological differences in embryo development, maternal obesity and aging perturb the mitochondrial DNA copy number at the blastocyst stage.

## 4. Discussion

This study presented new data on mitochondrial membrane potential and mtDNA kinetics during pre-implantation development, demonstrating cell-specific changes and differential responsiveness to diverse types of oxidative stress. The mtDNA copy number increased modestly between the 8-cell and blastocyst stages, and at this stage was not different between ICM and TE cells nor influenced by culture in optimized clinical-grade conditions compared to in vivo conceived embryos. In contrast, MMP appeared dynamic during in vitro culture between the 8-cell and blastocyst stages, was markedly increased in abnormal embryos, and exhibited cell-specific alterations in response to high oxygen (20% O_2_) exposure.

Compared to E2.5 embryos that went on to form morphologically normal blastocysts, those that developed abnormally or arrested contained mitochondria with higher MMP that were more active for a prolonged period. This suggests that the abnormal embryos upregulate mitochondrial function to compensate for underlying developmental defects. Alternatively, poor development could be due to the elevated MMP and associated production of cytotoxic ROS. That the best-quality embryos had relatively lower mitochondrial activity is in line with the ‘quiet metabolism’ hypothesis, which indicates that a low metabolic rate is optimal for healthy pre-implantation embryo development [38,39].

To better understand how diverse types of stress influence the mtDNA copy number in the blastocyst ICM, four different experimental models, which each exhibit increased oocyte mtROS levels, were utilized. Three of the models involved induction of mitochondrial dysfunction during oocyte maturation and prior to fertilization. Specifically, rotenone exposure was performed prior to ovulation and IVF, with zygotes exhibiting a high level of mtROS, similar to those from mice with pre-conception obesity or reproductive aging. In each of these three experimental models, IVF and embryo culture were performed under identical conditions as the controls and each other, yet distinct differences were observed in blastocysts. On-time development proceeded relatively normally in each context; however, the blastocyst mtDNA copy number was upregulated with maternal obesity, downregulated with maternal aging, and not changed by rotenone exposure. These differences underscore that obesity and aging cause additional perturbations to oocyte developmental competence beyond increased mtROS; for example, high lipid content in oocytes from obese females [19]. On the other hand, the similarities between the aging and rotenone exposure models, i.e., increased mtDNA in 8-cell embryos, suggest that this phenotype is due to common defects in Complex I activity. Notably, our previous study found a reduced mtDNA copy number in blastocysts from obese female mice [19], a difference likely resulting from the different embryo culture conditions between studies, which further highlights the critical importance of the in vitro environment on the embryo phenotype.

In the fourth model, oxidative stress was triggered acutely from the time of fertilization by exposure to 20% O_2_ during IVF and maintained until the blastocyst stage. Continuous oxidative stress during this window prevented timely development in a proportion of embryos, and in those that did form blastocysts on time, the mtDNA copy number was upregulated in the ICM, while MMP was downregulated in the TE. Thus, embryos exposed to 20% O_2_ culture upregulated the ICM mitochondrial function and mtDNA between the morula and blastocyst stages, potentially as a compensatory mechanism to maintain ATP production and viability. In line with this, the mtDNA copy number is positively correlated with oxidative phosphorylation in other contexts [40,41]. Further, previous studies found that embryos cultured in high oxygen contained fewer mitochondria [22], which when paired with our observations of increased mtDNA suggests a reduction in mitochondrial fission. Importantly, a comparison of embryo phenotypes from all four models indicated that continuous mitochondrial dysfunction during pre-implantation development, at least in response to 20% O_2_ culture, causes more significant defects than mitochondrial dysfunction that is present prior to conception but followed by embryogenesis in optimized conditions.

Mitochondria are key for maintaining pluripotency and regulating differentiation [42]; therefore, severe mitochondrial dysfunction resulting in the premature upregulation of mtDNA replication in the ICM could be indicative of early loss of pluripotency in these cells and have detrimental consequences for post-implantation development. In two of the experimental models, sufficient material permitted blastocyst embryo transfers to surrogates for gestation and analysis of fetal tissues. Interestingly, embryos cultured at 20% O_2_ were less likely to successfully implant and initiate fetal development, while embryos generated from rotenone-exposed oocytes were not compromised. This direct comparison between the two models again highlights that continuous oxidative stress throughout pre-implantation development impairs embryo viability to a greater extent than targeted induction of mtROS prior to fertilization. These preclinical findings support the current clinical standards that recommend culture of embryos in 6% CO_2_ and 5% O_2_ [43]. Further, in the embryos that implanted and grew to E18, the mtDNA copy number in fetal tissue was altered by high-oxygen culture during pre-implantation and by preconception rotenone exposure. Surprisingly, the mtDNA per cell was only altered in one of the two fetal tissues examined for each, and the direction of change did not match the relative levels detected in ICM of separate cohorts. Thus, it is likely that other compensatory mtDNA replication mechanisms are at play during gestational organogenesis, in addition to the well-established role of the mtDNA set-point determined in ICM [17].

In response to either maternal obesity or aging (the two physiological models of oocyte mitochondrial dysfunction), the blastocyst mtDNA content was altered, but this was not manifested in the ICM, suggesting alterations within the trophectoderm cell population. Even though all embryos were morphologically identical, it is possible that there were subtle differences in trophectoderm cell number. To our knowledge, the effects of maternal obesity and age on TE proliferation and/or differentiation have not been carefully examined in mouse models where confounders can be minimized. However, embryos of obese women (BMI > 30) were observed to have fewer cells (particularly trophectoderm [44]), while blastocysts from women of advanced age were reported to have normal ICM/TE organization [45]. Alternatively, the differences may be independent of the cell number and stem from premature induction of mtDNA replication in TE of obese blastocysts, and/or delayed mtDNA replication in embryos from aged females. Unfortunately, TE cells could not be analyzed directly because they are lysed during ICM immuno-purification and, thus, further work is needed to definitively determine whether obesity and aging exert differential defects on mtDNA replication and/or proliferation within the pre-implantation trophectoderm.

A better understanding of mtDNA kinetics in pre-implantation embryos is particularly relevant because the mtDNA copy number has been considered as a potential marker of human embryo quality [46,47]. Associations between the embryo mtDNA copy number and implantation potential have been uncovered in some studies but not others [48,49,50,51,52,53]. Importantly, mtDNA is measured in TE biopsies, which our preclinical data confirmed is not necessarily reflective of the mtDNA copy number within the ICM but may be important for successful implantation. Thus, it is essential for future research to investigate the impact of mitochondrial dysfunction in human oocytes on the blastocyst TE and ICM mtDNA content to elucidate the underlying biology and develop robust diagnostics. This will build on preclinical data to generate a better understanding of how these early events regulate mtDNA inheritance, and potentially influence offspring phenotypes.

## Data Availability

The data supporting the conclusions of this article will be made available by the authors upon reasonable request.

## Supplementary Materials

The following supporting information can be downloaded at: https://www.mdpi.com/article/10.3390/genes15030367/s1, Figure S1: Individual oocyte and embryo lysis and qPCR workflow. Figure S2: mtDNA copy number regulation across pre-implantation embryogenesis is relatively conserved between mouse strains. Figure S3: Embryo sex does not influence the blastocyst or ICM relative mtDNA content. Figure S4: TMRM signal is responsive to mitochondrial uncoupling and toxicants. Figure S5: On-time embryo development in aged and obese female mice.
