# Supplementary material for: Dynamics of Mitochondrial DNA Copy Number and Membrane Potential in Mouse Pre-Implantation Embryos: Responses to Diverse Types of Oxidative Stress
Source: Genes (Basel). 2024 Mar 16;15(3):367. doi: 10.3390/genes15030367 (PMC10970549; doi:10.3390/genes15030367)
Supplement: Supplementary file 1 [file genes-15-00367-s001.zip › genes-2880780-supplementary.pdf]

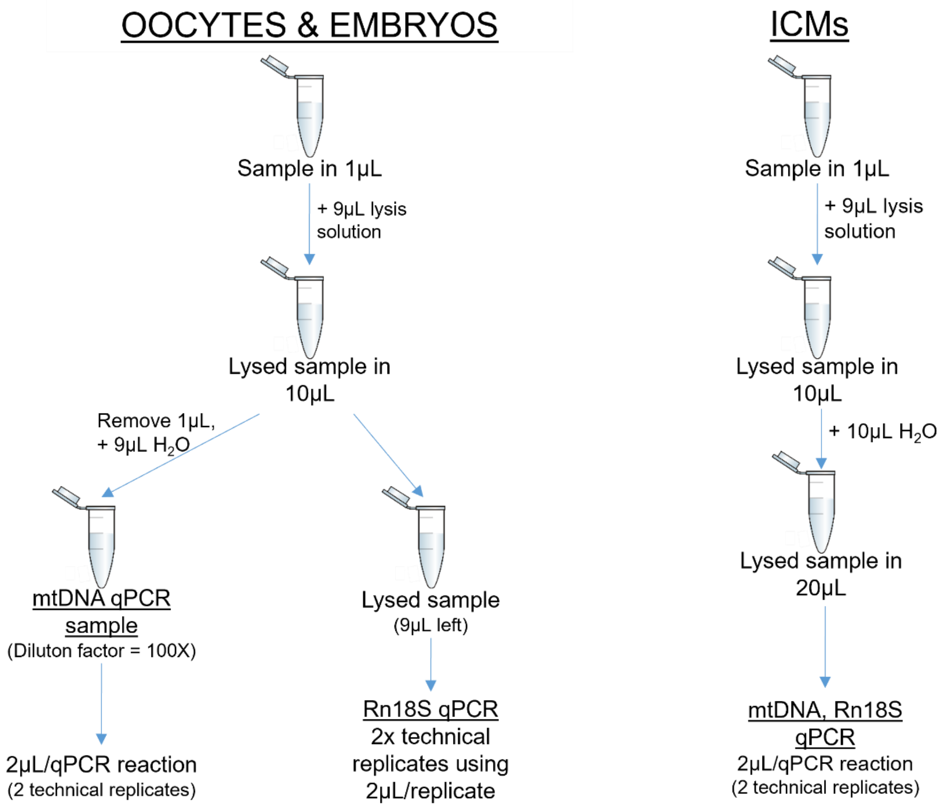

Figure S1. Individual oocyte and embryo lysis and qPCR workflow. Individual oocytes, cleavage stage embryos (2-cell, 4-cell, 8-cell, morula) and blastocysts were individually collected in 1µL sterile PBS-PVP, snap frozen and stored at -80^{0}C until use. To lyse cells and release DNA, 9µL of lysis solution was added, bringing the total sample volume to 10µL. Due to the relative abundance of mtDNA in oocytes and embryos, 1µL of lysis product was removed and further diluted in 9µL of sterile water to be used for mtDNA analysis (bringing the mtDNA dilution factor to 100X). The remaining 9µL of sample was used to complete 2 technical replicates each using Rn18S primers. After lysis, ICM samples were further diluted in 10µL of sterile water for analysis.

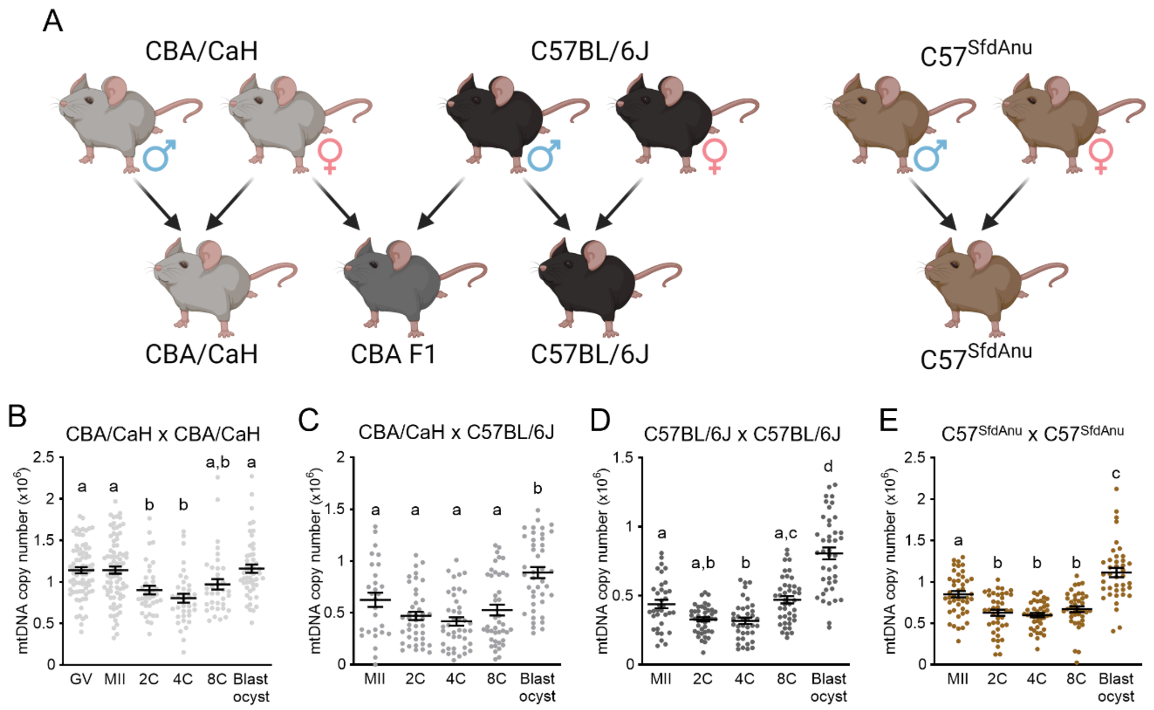

Figure S2. mtDNA copy number regulation across preimplantation embryogenesis is relatively conserved between mouse strains. (**A**) CBA/CaH female mice are crossed to C57BL/6J male mice to produce CBA F1 mice while C57^{SfdAnu} are an outbred strain with a C57BL/6J background. Total mtDNA copy number throughout a pre-implantation embryo development time course in (**B**) CBA/CaH x CBA/CaH (inbred) embryos; (**C**) CBA/CaH x C57BL/6J (hybrid) embryos; (**D**) C57BL/6J x C57BL/6J (inbred) embryos; and (**E**) C57^{SfdAnu} x C57^{SfdAnu} (outbred) embryos. N≥ 31 oocytes or embryos analyzed per strain per timepoint. Data analyzed using a linear mixed-effects model where different lowercase letters indicate statistical significance between groups of at least $p<0.05$.

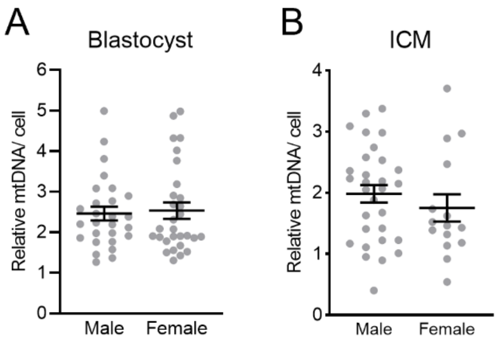

Figure S3. Embryo sex does not influence blastocyst or ICM relative mtDNA content. (**A**) Relative mtDNA content in male (n=27) and female (n=29) blastocysts. (**B**) Relative mtDNA content in the ICM of male (n=30) and female (n=15) blastocysts. Data analyzed using unpaired t-test.

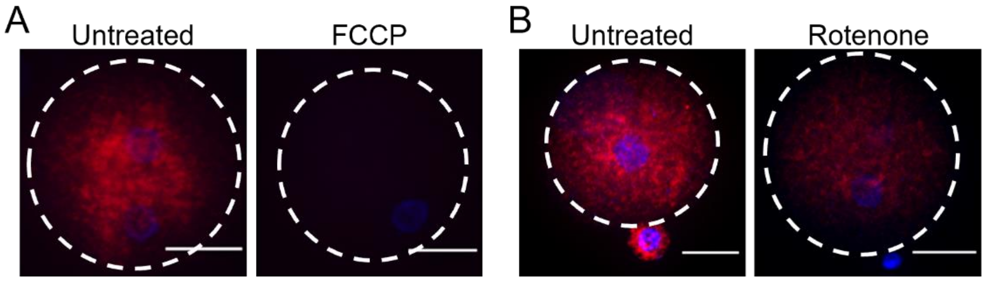

Figure S4. TMRM signal is responsive to mitochondrial uncoupling and toxicants. (**A**) To demonstrate the specificity of TMRM (red) as an indicator of MMP, after TMRM incubation (with Hoechst-3342 DNA stain (blue)), zygotes were exposed to 1μM FCCP for 10 minutes prior to imaging. FCCP-mediated uncoupling abrogated the TMRM/MMP signal. (**B**) Exposure of mice to a low dose of rotenone to disrupt Complex I function disrupted zygote TMRM signal. Images representative of 15-20 zygotes per group.

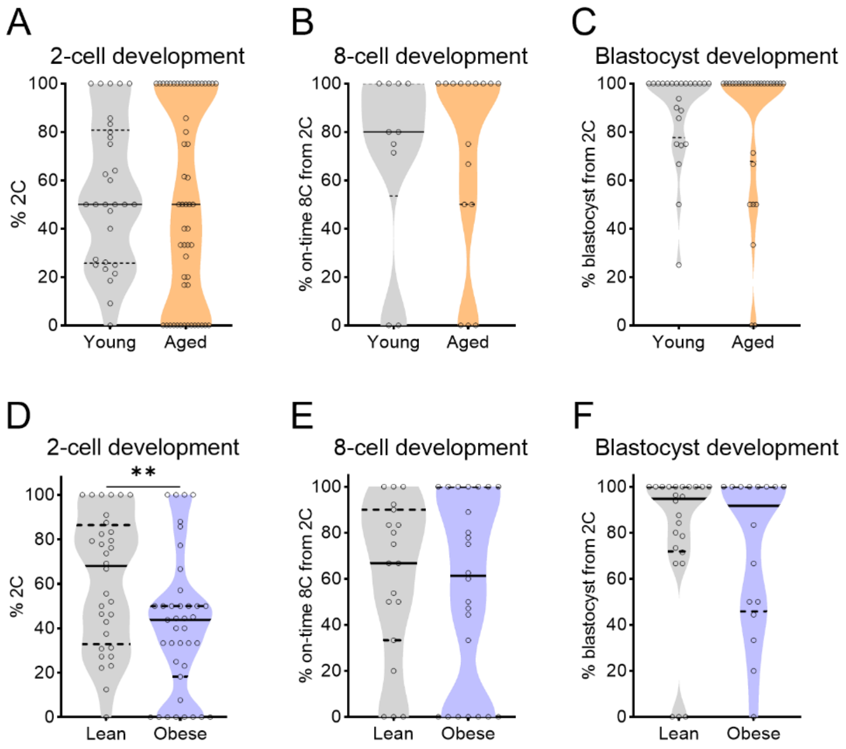

Figure S5. On-time embryo development in aged and obese female mice. On-time development to 2-cells (**A**), 8-cells (**B**) and blastocyst (**C**) following fertilization of oocytes from young or reproductively aged females. N=30 young and n=54 aged females. On-time embryos development to 2-cells (**D**), 8-cells (**E**) and blastocyst (**F**) following fertilization of oocytes from lean or obese females. N=33 lean and n=39 obese females.
